# Supplementary material for: Adaptability and agronomic performance evaluation of mungbean (Vigna radiata (L.) Wilczek) varieties under non-inoculated and inoculated rhizobium bacteria conditions
Source: PeerJ. 2025 Jun 16;13:e19558. doi: 10.7717/peerj.19558 (PMC12178244; doi:10.7717/peerj.19558)
Supplement: Supplemental Information 2 [file peerj-13-19558-s002.docx]

# Appendix TaBLES

Appendix Table 1. Levene's Test for Homogeneity of Variance for days to 50% flowering (DF), days to 90% physiological maturity (DM), number of nodules per plant (NNPP), nodule fresh weight per plant (NFWP), stand count (SC), plant height (PH), number of primary branches per plant (NPBP), number of pods per plant (NPPP), biological yield (BY), hundred seed weight (HSW), seed yield (SY), and harvest index (HI)

| **Grouping variable** | **F values for Levene’s test** | | | | | | | | | | | | |
| --- | --- | --- | --- | --- | --- | --- | --- | --- | --- | --- | --- | --- | --- |
|  | DoF | DF | DM | NNPP | NFWP | PH | NPBPP | NPPP | SC | HSW | BY | SY | HI |
| Year | 1 | 0.01^ns^ | 0.05^ns^ | 2.07^ns^ | 1.78^ns^ | 3.68^ns^ | 0.81^ns^ | 3.97^ns^ | 0.13^ns^ | 0.07^ns^ | 12.39^***^ | 8.89^**^ | 0.28^ns^ |

Appendix Table 2. The combined mean square values for days to 50% flowering, days to 90% physiological maturity, number of nodules per plant, fresh weight of nodules per plant, stand count, plant height, hundred seed weight and harvest index of mungbean as influenced by the main and interaction effects of varieties, Rhizobium bacteria inoculation levels and years

| **Source of variation** | **DoF** | **Combined mean squares** | | | | | | | | | |
| --- | --- | --- | --- | --- | --- | --- | --- | --- | --- | --- | --- |
|  |  | **DF (days)** | **DM (days)** | **NNPP (no)** | **FWNPP (no)** | **SC (no)** | **PH (cm)** | **NPBPP (no)** | **NPPPP (no)** | **HSW (g)** | **HI (%)** |
| Year | 1 | 58.02^***^ | 52.27^***^ | 26.67^***^ | 0.13^***^ | 1.3^ns^ | 40.02^*^ | 8.21^***^ | 88.33^***^ | 0.0167^ns^ | 765.38^***^ |
| Variety | 4 | 388.18^***^ | 617.23^***^ | 46.92^***^ | 0.23^***^ | 3561.6^***^ | 24.26^*^ | 2.27^***^ | 51.12^***^ | 6.4877^***^ | 107.45^***^ |
| Inoculation | 1 | 40.02^***^ | 77.07^***^ | 1.54^***^ | 0.02^**^ | 22.8^ns^ | 6.80^ns^ | 7.63^***^ | 2.09^ns^ | 0.0427^ns^ | 1.47^ns^ |
| Block: Year | 4 | 0.27^ns^ | 0.12^ns^ | 0.07^ns^ | 0.0006^ns^ | 203.6^*^ | 10.05^ns^ | 0.13^ns^ | 1.18^ns^ | 0.0087^ns^ | 5.14^ns^ |
| Variety: Year | 4 | 0.27^ns^ | 0.56^ns^ | 1.39^***^ | 0.007^*^ | 40.4^ns^ | 2.17^ns^ | 0.24^ns^ | 3.85^*^ | 0.0096^ns^ | 3.13^ns^ |
| Inoculation: Year | 1 | 0.02^ns^ | 2.40^ns^ | 0.60^**^ | 0.008^*^ | 212.8^ns^ | 9.76^ns^ | 0.003^ns^ | 2.24^ns^ | 0.006^ns^ | 8.10^ns^ |
| Variety: Inoculation | 4 | 2.68^*^ | 8.11^***^ | 0.25^**^ | 0.001^ns^ | 145.7^ns^ | 21.94^ns^ | 0.86^***^ | 4.90^*^ | 0.10^ns^ | 5.44^ns^ |
| Variety: Inoculation: Year | 4 | 0.27^ns^ | 0.11^ns^ | 0.07^ns^ | 0.001^ns^ | 48.7^ns^ | 16.24^ns^ | 0.19^ns^ | 2.51^ns^ | 0.01^ns^ | 1.64^ns^ |
| Residuals | 36 | 1.01 | 1.15 | 0.05 | 0.002 | 57.6 | 8.89 | 0.14 | 1.30 | 0.04 | 4.29 |
| CV (%) | | 2.52 | 1.28 | 4.40 | 11.00 | 7.73 | 10.13 | 5.84 | 9.51 | 4.59 | 8.02 |

Appendix Table 3. Mean square values of biological and seed yield of mungbean as influenced by the main and interaction effects of varieties and Rhizobium bacteria inoculation levels for the experiment conducted in 2021 and 2022

| **Source of variation** | **DoF** | **Mean squares for 2021 year** | | **Mean squares for 2022 year** | |
| --- | --- | --- | --- | --- | --- |
|  |  | **Biological yield** | **Seed yield** | **Biological yield** | **Seed yield** |
| Block | 2 | 298775^*^ | 34120^*^ | 6711^ns^ | 3117^ns^ |
| Variety | 4 | 2571733^***^ | 356613^***^ | 424546^**^ | 65544^***^ |
| Inoculation | 1 | 714718^**^ | 66458^*^ | 47336^ns^ | 715^ns^ |
| Variety: Inoculation | 4 | 204325^*^ | 37279^*^ | 59975^ns^ | 2794^ns^ |
| Residuals | 18 | 65245 | 8935 | 42624 | 6899 |
| CV (%) | | 8.01 | 9.96 | 8.80 | 15.79 |
